# Sailing Across Contraception, Pregnancy, and Breastfeeding: The Complex Journey of Women with Cardiomyopathies

**DOI:** 10.3390/jcm14144977

**Published:** 2025-07-14

**Authors:** Maria Cristina Carella, Vincenzo Ezio Santobuono, Francesca Maria Grosso, Marco Maria Dicorato, Paolo Basile, Ilaria Dentamaro, Maria Ludovica Naccarati, Daniela Santoro, Francesco Monitillo, Rosanna Valecce, Roberta Ruggieri, Aldo Agea, Martino Pepe, Gianluca Pontone, Antonella Vimercati, Ettore Cicinelli, Nicola Laforgia, Nicoletta Resta, Andrea Igoren Guaricci, Marco Matteo Ciccone, Cinzia Forleo

**Affiliations:** 1 Cardiology Unit, Department of Interdisciplinary Medicine (DIM), University of Bari “Aldo Moro”, University Hospital Consortium Polyclinic of Bari, 70124 Bari, Italy; m.carella31@phd.uniba.it (M.C.C.); vincenzoezio.santobuono@uniba.it (V.E.S.); m.dicorato20@studenti.uniba.it (M.M.D.); paolo.basile@uniba.it (P.B.); ilaria.dentamaro@gmail.com (I.D.); marialudovica97@libero.it (M.L.N.); danina2012@gmail.com (D.S.); fmonitillo@libero.it (F.M.); aldo.agea@gmail.com (A.A.); martino.pepe@uniba.it (M.P.); andreaigoren.guaricci@uniba.it (A.I.G.); marcomatteo.ciccone@uniba.it (M.M.C.); 2 Internal Medicine Section, Department of Precision and Regenerative Medicine and Ionian Area (DiMePRe-J), University of Bari “Aldo Moro”, University Hospital Consortium Polyclinic of Bari, 70124 Bari, Italy; francesca.grosso@asst-fbf-sacco.it; 3 Functional Department of Prevention, ASST Fatebenefratelli Sacco, 20133 Milan, Italy; 4 UOC Cardiologia, Ospedale Di Venere, Asl Bari, 70124 Bari, Italy; valeccer@gmail.com (R.V.); roberta.ruggieri1990@gmail.com (R.R.); 5 Department of Perioperative Cardiology and Cardiovascular Imaging, Centro Cardiologico Monzino IRCCS, 20133 Milan, Italy; gianluca.pontone@cardiologicomonzino.it; 6 Department of Biomedical, Surgical and Dental Sciences, University of Milan, 20133 Milan, Italy; 7 Unit of Obstetrics and Gynecology, Department of Interdisciplinary Medicine (DIM), University of Bari “Aldo Moro”, University Hospital Consortium Polyclinic of Bari, 70124 Bari, Italy; antonella.vimercati@uniba.it (A.V.); ettore.cicinelli@uniba.it (E.C.); 8 Neonatology and Neonatal Intensive Care Unit, Department of Interdisciplinary Medicine (DIM), University of Bari “Aldo Moro”, University Hospital Consortium Polyclinic of Bari, 70124 Bari, Italy; nicola.laforgia@uniba.it; 9 Medical Genetics Unit, Department of Precision and Regenerative Medicine and Ionian Area (DiMePRe-J), University of Bari “Aldo Moro”, University Hospital Consortium Polyclinic of Bari, 70124 Bari, Italy; nicoletta.resta@uniba.it

**Keywords:** pregnancy, breastfeeding, lactation, neonatology, cardiomyopathy, reproductive health, obstetrics, gynecology, women’s health, sexual health, gender differences

## Abstract

Gender-specific cardiology has gained increasing recognition in recent years, emphasizing the need for tailored management strategies for women with cardiovascular disease. Among these, cardiomyopathies—dilated, arrhythmogenic, hypertrophic, and restrictive—pose unique challenges throughout a woman’s reproductive life, affecting contraception choices, pregnancy outcomes, and breastfeeding feasibility. Despite significant advances in cardiovascular care, there is still limited guidance on balancing maternal safety and neonatal well-being in this complex setting. This review provides a comprehensive overview of the current evidence on reproductive counseling, pregnancy management, and postpartum considerations in women with cardiomyopathies. We discuss the cardiovascular risks associated with each cardiomyopathy subtype during pregnancy, highlighting risk stratification tools and emerging therapeutic strategies. Additionally, we address the safety and implications of breastfeeding, an often overlooked but increasingly relevant aspect of postpartum care. A multidisciplinary approach involving cardiologists, gynecologists, obstetricians, and anesthesiologists is crucial to optimizing maternal and fetal outcomes. Improved risk assessment, tailored patient counseling, and careful management strategies are essential to ensuring safer reproductive choices for women with cardiomyopathy. From now on, greater attention is expected to be given to bridging existing knowledge gaps, promoting a more personalized and evidence-based approach to managing these patients throughout different stages of reproductive life.

## 1. Introduction

It is well established that pregnancy instigates significant cardiovascular adaptations in women to meet the increased metabolic demands of the growing fetus. During the first two trimesters, maternal blood volume and cardiac output rise by approximately 30–50%, while resting heart rate increases by 10–15 beats per minute. A physiological myocardial hypertrophy and plasma volume expansion result in relative hemodilution and reduced systemic vascular resistance [1]. Although these changes are usually well tolerated by healthy women and tend to reverse within six months postpartum, they may unmask or exacerbate underlying myocardial disease in predisposed individuals [2,3,4].

Cardiomyopathies represent a particular risk during pregnancy. These conditions may be clinically silent or mildly symptomatic before gestation, but can manifest with heart failure, arrhythmias, or hemodynamic collapse due to the circulatory burden imposed by pregnancy. Notably, cardiomyopathy-related symptoms can first appear during gestation, acting as a clinical trigger for the diagnosis. Spontaneous abortion occurs in approximately 15% of these women, and cardiovascular disease now accounts for up to 26% of all pregnancy-related maternal deaths [1,5,6,7].

**Figure 1:**
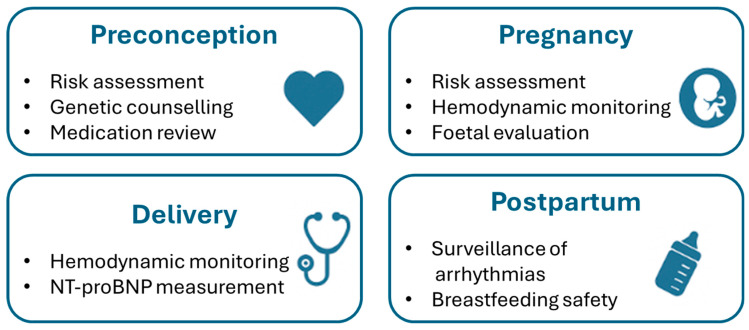
Reproductive journey of women with cardiomyopathy.

Despite growing awareness, structured cardio-obstetric protocols are still lacking in many settings. This review aims to synthesize current evidence on the management of women with cardiomyopathies during the reproductive journey, from contraception to pregnancy and breastfeeding, highlighting the need for individualized, multidisciplinary strategies and early risk stratification to improve both maternal and neonatal outcomes (Figure 1).

## 2. Methods

We performed a comprehensive literature search across Ovid MEDLINE, Scopus, and Web of Science (WOS) databases to identify studies published up to April 2025. The search aimed to capture evidence on the reproductive management—including contraception, pregnancy, and breastfeeding—of women with cardiomyopathies, encompassing dilated, hypertrophic, arrhythmogenic, and restrictive phenotypes.

Inclusion criteria comprised original observational studies, clinical registries, case reports, and international guidelines that specifically reported maternal and/or fetal outcomes, risk stratification approaches, genetic counseling considerations, or multidisciplinary management recommendations in these patient populations. Exclusion criteria were studies focused exclusively on peripartum cardiomyopathy without relevance to other cardiomyopathy phenotypes, non-human data, and non-original work unless offering expert consensus or guideline recommendations.

While this is a narrative review and not a systematic review, we sought to enhance transparency by applying structured selection criteria and by thematically synthesizing evidence consistent with best practices for narrative reviews. We did not apply PRISMA guidelines in full but followed its spirit of methodological clarity.

We also deliberately included both original studies and case reports, acknowledging the heterogeneity and relative rarity of cardiomyopathy subtypes in pregnancy. Case reports, although weighted less heavily in our interpretation, were included to illustrate specific clinical challenges, rare phenotypes, and management considerations not always captured in larger cohort studies.

The initial search yielded 84 results from Scopus, 546 from WOS, and 236 from Ovid. After removing duplicates, 55 articles were selected as most relevant for inclusion. Of these, 23 originated from high-income countries. The final selection comprised 47 original research articles, 2 clinical guidelines or consensus documents, 2 narrative reviews, 2 systematic reviews, and 2 case reports. Data were charted and synthesized thematically by the authors with multidisciplinary input from experts in cardiology, gynecology, obstetrics, neonatology, and clinical genetics.

## 3. Cardiomyopathies in Women: From Pathophysiology to Reproductive Challenges

Cardiomyopathies represent a heterogeneous group of myocardial disorders characterized by structural and functional abnormalities not explained by abnormal loading conditions or coronary artery disease [8]. While traditionally studied in general populations, mounting evidence has underscored the necessity for sex-specific methodologies in the diagnosis and management of these conditions, particularly in the context of women’s reproductive health [9]. Indeed, the interaction between cardiomyopathy and key reproductive milestones—contraception, pregnancy, and breastfeeding—presents unique clinical challenges that require personalized strategies, multidisciplinary coordination, and a nuanced understanding of disease biology in women.

Among the different subtypes of cardiomyopathies—hypertrophic (HCM), dilated (DCM), arrhythmogenic (ACM/ARVC), and restrictive (RCM)—many are inherited in an autosomal dominant fashion, meaning women are equally likely to carry genetic mutations. However, penetrance and phenotype expression differ markedly by sex. For example, in conditions like ARVC and Lamin A/C gene (LMNA)-related DCM, men tend to exhibit earlier onset and more severe disease, but this does not equate to negligible risk in women, especially when compounded by physiological stressors such as pregnancy [10,11] (Table 1).

Pregnancy imposes a substantial hemodynamic burden on the cardiovascular system, with a 30–50% increase in cardiac output, reduced systemic vascular resistance, and plasma volume expansion. In women with cardiomyopathies, these changes may unmask or exacerbate latent myocardial dysfunction, thereby predisposing to arrhythmias, acute heart failure, or even sudden cardiac death [1,7,12]. It is important to note that the transition from the late second trimester through the early postpartum period is considered a particularly high-risk window due to peak circulatory demands and abrupt shifts in fluid balance [13,14].

Until the last decade, clinical evidence on cardiomyopathies in pregnancy remained sparse and often anecdotal, with early studies either excluding pregnant women or grouping cardiomyopathies with vastly different pathophysiology [1,15]. This created a significant gap in the development of robust, evidence-based guidelines. Furthermore, much of the existing literature focused on peripartum cardiomyopathy (PPCM), the sole female-specific subtype, while inherited cardiomyopathies were often underreported or misclassified [1].

The increased availability of genetic testing has result in a growing number of asymptomatic female carriers being identified during reproductive age. This has brought into sharp relief the necessity for integrated reproductive and cardiovascular counseling, encompassing preconception planning, medication review, and inheritance risk assessment [11]. Moreover, given the risk of transmitting pathogenic variants to offspring, and the possibility, albeit rare, of phenotypic expression in the prenatal period or early childhood, preconception genetic counseling is essential. In this context, prenatal assessment strategies such as detailed fetal ultrasound or invasive diagnostic procedures (e.g., amniocentesis) may be considered to provide a more complete evaluation of potential fetal involvement.

Women remain significantly underrepresented in most cardiomyopathy trials, particularly in relation to therapeutic interventions, implantable cardioverter defibrillator (ICD) implantation, and disease progression modeling [9,16,17]. The evolving field of cardio-obstetrics has catalyzed a paradigm shift toward more structured, risk-guided care for women with cardiovascular conditions, including cardiomyopathies [8]. Recent guidelines and position papers now emphasize the importance of multidisciplinary teams, including cardiologists, maternal-fetal medicine specialists, anesthesiologists, and neonatologists, to optimize maternal and fetal outcomes [7,10,13]. This evolution is characterised by an increased awareness of sex-related differences in treatment response and pharmacokinetics, which may influence therapy during pregnancy and the postpartum period.

As a result, there is growing recognition that cardiomyopathy in women must be approached not as a static diagnosis but as a dynamic condition influenced by hormonal, hemodynamic, and genetic factors, particularly in the context of reproductive health.

### 3.1. Historical Perspectives and Risk Evolution in Pregnant Women with Cardiomyopathies

Historically, pregnancy in women with cardiomyopathies has been considered high risk, largely due to the hemodynamic stress it imposes and the potential for life-threatening cardiovascular complications. Nevertheless, the manner in which clinicians evaluate and manage this risk has evolved considerably over the past two decades. Early systematic reviews, such as the one by Krul et al. in 2011 [1], were among the first to aggregate data on the pregnancy outcomes in inherited cardiomyopathies, emphasizing that while many asymptomatic women tolerated pregnancy well, those with previous cardiac events, poor functional class (New York Heart Association, NYHA III–IV), or severely reduced left ventricular function faced markedly increased maternal risk [1,18]. The same review also highlighted the lack of long-term follow-up studies and called for structured preconception counseling and genetic evaluation.

A few years later, Van Tintelen and colleagues (2014) advanced this concept by introducing the relevance of genotype to pregnancy risk. Their work underscored how specific mutations—such as those in LMNA, Titin (TTN), or transmembrane protein 43 (TMEM43)—can influence both baseline risk and clinical deterioration during pregnancy, even in previously asymptomatic women [11]. This insight helped shift the clinical paradigm from symptom-based assessment alone to a more nuanced approach incorporating genotype-phenotype correlations. It was also observed that there were marked discrepancies in the penetrance and progression of the disease according to sex, with the emphasis placed on the necessity for a bespoke management approach for women of reproductive age.

The same year, Bonnet et al. published the NORMANDY study, a retrospective analysis of 197 pregnancies in women with various cardiac diseases, including cardiomyopathies, managed between 2000 and 2014 [13]. Maternal cardiovascular events occurred in 6.6% of cases, but complications were significantly more frequent among patients with cardiomyopathies compared to other structural heart conditions [13]. Obstetric and neonatal complications were also common, affecting roughly one-third of pregnancies [13]. Importantly, the study documented a transition toward multidisciplinary and individualized care, which correlated with improved maternal and fetal outcomes over time.

In a separate but complementary retrospective cohort from the Hôpital de la Pitié-Salpêtrière, Charron et al. (2018) [19] reported that 35% of pregnancies in women with cardiomyopathies were complicated by major cardiovascular events, including heart failure, arrhythmias, and even cardiac death [19]. Interestingly, three maternal deaths occurred in women who were not followed through the hospital’s structured multidisciplinary protocol, reinforcing the importance of early and coordinated management. The study also confirmed that dilated cardiomyopathy was associated with the highest maternal risk, and suggested that commonly used scores like modified WHO (mWHO) and ZAHARA were less predictive than CARPREG in this specific population [19,20,21,22,23].

Over time, further epidemiological analyses confirmed and refined these findings. Schaufelberger (2019) noted that while HCM is generally associated with favorable pregnancy outcomes, adverse events such as arrhythmias and heart failure may still occur, particularly in high-risk patients [14]. For ARVC, pregnancy-related complications, especially ventricular arrhythmias, were reported in up to one-third of cases, with syncope and preterm delivery as additional concerns. Evidence for restrictive and left ventricular non-compaction cardiomyopathy remained sparse, often limited to case reports.

Most recently, a large-scale U.S. analysis by Satti et al. (2024) [15] provided new insights into trends over a 15-year period, analyzing more than 37,000 delivery hospitalizations complicated by cardiomyopathy. Their data confirmed that postpartum cardiomyopathy (PPCM) remained the leading subtype in terms of frequency and severity, accounting for nearly 46% of cases and associated with the highest rates of in-hospital mortality and acute heart failure [15]. However, they also noted a relative increase in the prevalence of DCM and HCM over time, perhaps reflecting improved diagnostic capabilities and longer survival of women with cardiomyopathies into reproductive age [15].

Taken together, these studies reflect a shift from fragmented, anecdotal understanding to a more evidence-based, stratified approach to managing pregnancy in women with cardiomyopathy. However, they also underscore persistent limitations, such as small sample sizes, underrepresentation of rare subtypes, and limited long-term data, that continue to hamper optimal clinical decision-making. As more women with cardiomyopathies reach childbearing age thanks to advances in diagnosis and therapy, there is a pressing need for prospective, multicenter registries and inclusion of these populations in clinical trials. In addition to cardiomyopathy-specific risks, general obstetric complications such as gestational diabetes and hypertension should be routinely screened and managed according to standard pregnancy guidelines [5,24,25].

### 3.2. Risk Stratification in Pregnancy: Refining the Tools for Women with Cardiomyopathy

Accurate risk stratification remains a cornerstone in the management of pregnancy for women with cardiomyopathies, allowing clinicians to guide preconception counseling, tailor surveillance intensity, and inform delivery planning. However, the majority of extant risk models were initially designed for broader populations with congenital or acquired heart disease, and only more recently adapted or tested for cardiomyopathy-specific scenarios. Among the first widely adopted models, the mWHO classification provided a pragmatic, disease-based stratification system, dividing cardiovascular conditions into four classes of increasing maternal risk [20]. Despite its ease of application, the method has been the subject of criticism due to its limited granularity and reliance on expert consensus rather than prospective validation, particularly in the context of inherited or rare cardiomyopathies [7].

The CARPREG score, introduced in 2001, incorporated clinical variables such as prior cardiac events and baseline NYHA class [7,22,23,26]. However, its predictive value in women with cardiomyopathy was limited by underrepresentation of this subgroup in the original cohort. The more recent CARPREG II score aimed to address this limitation by integrating echocardiographic and hemodynamic parameters, such as left ventricular ejection fraction (LVEF) and the presence of mechanical valves, offering improved discrimination in high-risk patients [26]. In a focused study on inherited cardiomyopathies, Wallet et al. demonstrated that CARPREG II outperformed both mWHO and ZAHARA in predicting major cardiovascular events during pregnancy, with an AUC of 0.78 compared to 0.70 and 0.60, respectively [26]. This is particularly relevant in DCM, where risk is often underestimated, and in HCM or ARVC, where arrhythmogenic burden may not correlate with functional class alone. Still, no score perfectly predicts individual trajectories. Major cardiac events occurred even in patients with a CARPREG score of 0, highlighting the limits of current models when applied to rare genotypes or mild baseline disease [19]. Therefore, while risk scores remain useful for population-level guidance, individualized assessment, integrating genotype, arrhythmic burden, functional reserve, and patient values, remains essential (Table 2).

While these models offer practical guidance, their predictive accuracy in cardiomyopathy remains an area of ongoing investigation. Notably, recent work by Wallet et al. represents one of the first dedicated attempts to validate CARPREG II in inherited cardiomyopathies, suggesting improved discrimination but underscoring the need for broader, prospective studies [26]. In addition, emerging machine learning and AI-based approaches hold promise for refining risk prediction in pregnancy, though no such tools have yet been developed or validated specifically for women with cardiomyopathy.

## 4. Hypertrophic Cardiomyopathy and Pregnancy: Clinical Risks and Outcomes

HCM is a genetically determined cardiac disorder with variable phenotypic expression and clinical presentation, increasingly diagnosed in women of reproductive age due to familial screening and improved imaging modalities [27,28,29,30,31,32]. Pregnancy in HCM has historically been considered relatively safe, but accumulating evidence reveals a more nuanced picture, with potential for cardiovascular and obstetric complications, particularly in the presence of high-risk features such as left ventricular outflow tract obstruction (LVOTO), arrhythmias, or functional limitation.

Preliminary studies suggested a favorable maternal prognosis, especially in asymptomatic women. Most women with HCM can tolerate pregnancy well, although complications such as heart failure and arrhythmias were not negligible, particularly in those with prior symptoms [33]. These findings were echoed in a large retrospective cohort by Fumagalli et al., which analyzed 432 pregnancies in 242 women with HCM and found that pregnancy was not associated with worse long-term outcomes. In fact, in multivariate analysis, pregnancy was linked to a lower risk of major adverse cardiovascular events (HR 0.605, 95% CI 0.380–0.963, *p* = 0.034). Peripartum complications occurred in fewer than 8% of cases and were mainly observed in women with a known diagnosis and symptomatic disease [34].

The ROPAC registry (Registry of Pregnancy and Cardiac Disease) provided key insights into HCM specifically [35]. In its dedicated HCM analysis, Goland et al. included 60 women, 42% of whom had obstructive disease [35]. Although no maternal mortality was observed, 23% experienced major adverse cardiovascular events (MACE), primarily heart failure and ventricular arrhythmias. Notably, events clustered in the third trimester and early postpartum period. Importantly, there was no statistically significant difference in MACE between obstructive and non-obstructive forms, though the authors noted that the study might have been underpowered to detect such differences [35].

In a nationwide population-based study using Korean National Health Insurance data, Choi et al. identified 158 pregnancies in 122 women with HCM and reported cardiovascular events in 14 women (8.8%), including heart failure and both new-onset atrial fibrillation and ventricular tachycardia [36]. Pre-existing arrhythmias were the strongest predictors of adverse outcomes, with odds ratios of 7.44 and 31.61, respectively [36]. Similarly, in a Chinese cohort of 100 women with HCM, Huang et al. identified obstructive HCM, NYHA class ≥ II, and a history of syncope as independent predictors of cardiac complications [37]. The maternal complication rate included 2% mortality and 7% incidence of heart failure, with most events occurring in the third trimester and postpartum [37].

Case reports further illustrate the complexity of pregnancy in HCM. Alves-Pinto described a patient with severe LVOTO (peak gradient 90 mmHg) who required preterm delivery at 34 weeks due to fetal growth restriction, despite preserved maternal stability [38]. Other reports describe LVOTO being first diagnosed during pregnancy, often triggered by increased hemodynamic demands and resulting in sustained arrhythmias, ICD implantation, or postpartum septal reduction therapy [39,40,41].

Importantly, recent data also suggest that pregnancy itself may not worsen the natural history of HCM. Fumagalli et al., in a longitudinal follow-up, found that women who experienced pregnancy did not have an increased incidence of disease progression, ICD therapy, or sudden cardiac death compared to nulligravid controls [34]. Indeed, the pregnancy group exhibited a reduced hazard of composite cardiovascular outcomes, raising hypotheses about favorable selection or protective hormonal environments.

Taken together, these data confirm that pregnancy is generally well tolerated in women with HCM, particularly in those who are asymptomatic, normotensive, and without significant LVOTO or arrhythmias. However, MACE rates range from 10% to 25% across cohorts, underscoring the need for thorough preconception evaluation (Table 3). The third trimester and early postpartum remain the periods of highest vulnerability. Pre-pregnancy risk stratification and individualized multidisciplinary management are therefore essential for optimizing maternal and fetal outcomes.

### 4.1. Obstructive vs. Non-Obstructive HCM: Prognosis and Management During Pregnancy

Pregnancy in HCM presents unique clinical considerations that are largely dictated by the presence or absence of dynamic LVOTO. Beyond prognostic implications, which have been addressed elsewhere, the distinction between obstructive (OHCM) and non-obstructive (NOHCM) forms carries significant weight in the clinical management, pharmacologic strategy, and delivery planning of affected women.

In OHCM, the reduction in systemic vascular resistance and the increase in heart rate that characterize pregnancy physiology can exacerbate the outflow gradient, leading to impaired ventricular filling, worsening diastolic dysfunction, and elevated risk of exertional symptoms or decompensation during labor [37,40,42]. These hemodynamic changes are particularly relevant in the third trimester and peripartum period, when cardiac preload varies rapidly and can unmask latent obstruction [42].

Therapeutic approaches in OHCM require proactive modulation of loading conditions. Beta-blockers remain the mainstay of treatment, with proven safety in pregnancy and the capacity to blunt adrenergic response, reduce LVOT gradients, and promote diastolic filling [43]. In refractory cases, verapamil may be considered, though hypotension and negative inotropy must be carefully monitored. Disopyramide, while effective at reducing obstruction, is generally avoided due to limited safety data in pregnancy.

For patients with severe gradients and symptoms, pre-pregnancy septal reduction therapy (SRT) may be a valuable option. A case by Tomasov et al. described successful pregnancy following alcohol septal ablation (ASA) performed prior to conception, with complete resolution of obstruction and no maternal-fetal complications [41]. This approach may be advisable in women with LVOTO ≥ 50 mmHg at rest and refractory symptoms despite optimal medical therapy. Notably, ASA should be performed well in advance of pregnancy to allow stabilization of scar remodeling.

**Figure 2:**
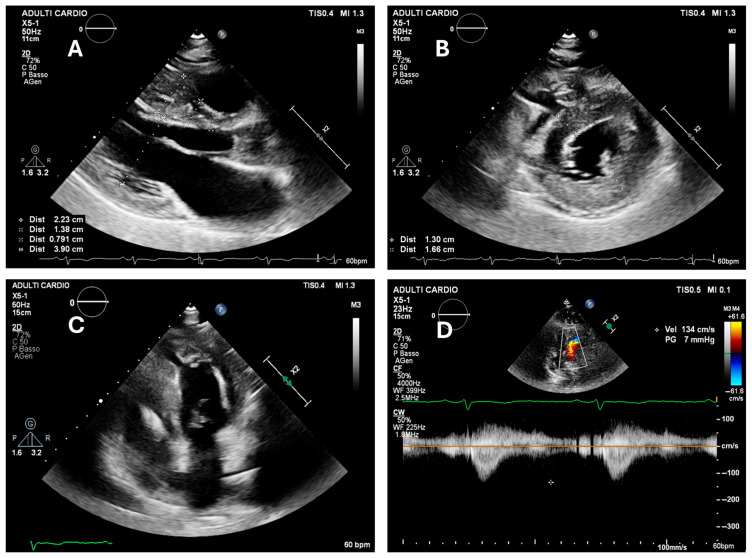
Transthoracic echocardiogram from a young pregnant woman with non-obstructive hypertrophic cardiomyopathy. (**Panel A**): Parasternal long-axis view showing marked septal hypertrophy. (**Panel B**): Parasternal short-axis view at mid-ventricular level, demonstrating severe hypertrophy of the inferolateral wall. (**Panel C**): Aneurysmal appearance of the true apex, with mildly reduced global systolic function due to diffuse hypokinesia. (**Panel D**): No significant intraventricular gradient detected at rest or following the Valsalva maneuver (* indicates a peak gradient of 7 mmHg).

In contrast, women with NOHCM typically have more preserved hemodynamic profiles and require a different approach. In these cases, the focus shifts from gradient management to monitoring of diastolic function and arrhythmic risk. Women with impaired relaxation, enlarged left atrium, or prior atrial fibrillation may decompensate during pregnancy even in the absence of obstruction [35]. In such cases, rhythm control strategies and tailored volume management become essential (Figure 2).

Another critical difference lies in delivery planning. In OHCM, cesarean section may be favored in cases of severe obstruction, especially when associated with hypotension or arrhythmias. However, for well-compensated NOHCM patients, vaginal delivery under epidural anesthesia remains a safe and preferred option. Hemodynamic monitoring during labor is recommended in both groups, though continuous telemetry is especially crucial in OHCM to detect arrhythmic events early [40,44].

Finally, ICD carriers, more common among women with OHCM and high-risk profiles, do not automatically require cesarean section, but peripartum magnet deactivation and immediate post-delivery reprogramming should be coordinated with electrophysiology teams [45].

In summary, OHCM and NOHCM require fundamentally different management strategies during pregnancy. While both may tolerate gestation under careful surveillance, the presence of LVOTO warrants earlier intervention, tighter hemodynamic control, and possible SRT before conception. Tailored delivery planning and multidisciplinary coordination remain the cornerstone of optimal care.

### 4.2. Preconception Counselling and Risk Stratification in Women with Hypertrophic Cardiomyopathy

Preconception counselling is widely recommended as part of care for women with HCM planning pregnancy [5,8,27]. While many tolerate gestation well, individual risk assessment is essential to anticipate cardiovascular complications, tailor monitoring, and define delivery strategies. The heterogeneity of HCM expression, in terms of obstruction, arrhythmias, and genetic background, demands a personalized, multidisciplinary approach, ideally involving cardiology, maternal-fetal medicine, electrophysiology, and anesthesiology specialists [31,32].

Functional status remains the most reproducible and clinically meaningful predictor. Fumagalli et al. showed that women with NYHA class I before conception had significantly fewer cardiovascular events than those in class II or higher, with NYHA class III–IV representing a major risk factor for maternal MACE (HR 5.29, *p* < 0.001) [34]. Structural and hemodynamic markers such as left atrial enlargement, LVOT gradient, and LV wall thickness also contribute to prognostication. In one study, an LVOT gradient > 53.5 mmHg had 90.9% sensitivity and 73.5% specificity for predicting MACE [42]. Conversely, in another cohort, LVOTO alone did not predict adverse maternal outcomes unless accompanied by symptoms or arrhythmias [19]. This discrepancy underscores the need to integrate imaging findings with clinical context.

Several risk classification models have been proposed for pregnant women with cardiovascular disease, but their accuracy in HCM remains under scrutiny. The modified WHO classification is the most widely endorsed, placing HCM in class II or III depending on phenotype and symptom burden [46]. The CARPREG II score, a composite tool including lesion-specific and general risk factors, has shown promising discrimination in women with inherited cardiomyopathies. In a dedicated analysis of 90 pregnancies (46 with HCM), a CARPREG II score ≥ 4 identified a 50% risk of cardiac events, while a score of 0–1 correlated with only 5.5% event rate [26]. Notably, the CARPREG II outperformed the original CARPREG and mWHO models in predicting complications in this population, with an AUC of 0.782.

Another critical issue during counselling is timing of pregnancy in relation to diagnosis and disease stability. In a recent cohort, many women became pregnant before being diagnosed with HCM, which led to a delay in risk stratification and, in some cases, adverse outcomes [44]. Therefore, timely identification and comprehensive pre-pregnancy evaluation, including echocardiography, Holter monitoring, exercise tolerance, and, where relevant, genetic counselling, are essential.

## 5. Epidemiology and Heart Failure Risk in Pregnant Women with Dilated Cardiomyopathy

DCM represents a high-risk clinical condition during pregnancy due to the significant hemodynamic changes that occur throughout gestation [47,48]. Several recent studies have investigated the safety of pregnancy in women with genetically determined DCM and in carriers of pathogenic variants without a manifest phenotype at the time of conception [49]. One of the most consistent findings is that pregnancy may act as a ‘second hit’ in genetically predisposed individuals (according to Knudson’s hypothesis), triggering the onset of the clinical phenotype [49].

In the largest multicenter study available to date, conducted on 48 women carrying genetic variants associated with DCM, 31% experienced adverse cardiac events during pregnancy or within six months postpartum, with a prevalence of 50% among women with known DCM and 17% among asymptomatic carriers [50]. Reported events included episodes of acute heart failure, sustained ventricular tachycardia, the need for left ventricular assist device implantation, heart transplantation, and one case of maternal death. Predictors of adverse outcomes included a history of pre-pregnancy cardiac events and pre-existing moderate left ventricular dysfunction (LVEF < 45%) [50].

The experience reported by Peters et al. supports these findings, highlighting that although most women with pathogenic DCM variants tolerated pregnancy well, approximately 10% developed serious complications, including one case of cardiac arrest and intra-gestational death in a patient with a TTNtv variant [51]. This risk appears comparable to that documented in women with pre-existing structural heart disease.

In another prospective study conducted in Israel, seven women with known DCM or diagnosed during the first trimester were followed throughout pregnancy and up to 18 months postpartum [52]. Despite stable echocardiographic parameters (with a mean LVEF consistently around 35%), three patients (42%) experienced significant complications: two episodes of acute heart failure and one case of massive pulmonary embolism. An early and significant increase in plasma NT-proBNP levels was found to be predictive of adverse events, suggesting the usefulness of this biomarker in clinical monitoring [52,53].

Overall, although many women with stable DCM and NYHA class I–II can undergo pregnancy without major adverse events, the cumulative risk of cardiac complications remains high, particularly within the first six months postpartum. Poor prognostic indicators include an ejection fraction < 30%, a history of arrhythmic events, biventricular dilation, and NYHA class ≥ III [6].

**Figure 3:**
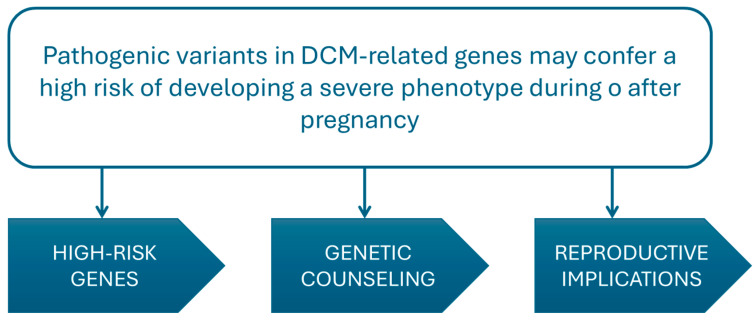
Genetic considerations in pregnancy associated with dilated cardiomyopathy.

In summary, pregnancy in women with DCM, especially of genetic origin, requires thorough preconception evaluation, close multidisciplinary follow-up, and continuous monitoring throughout the postpartum period (Figure 3). The combined use of clinical assessment, echocardiographic evaluation, and biomarkers such as NT-proBNP may enhance early identification of patients at risk for hemodynamic deterioration.

### 5.1. Fetal and Neonatal Outcomes in Pregnancies Complicated by Dilated Cardiomyopathy

The impact of DCM on fetal and neonatal health is significant and closely linked to maternal hemodynamic stability during pregnancy and delivery. Available data indicate that, although some women with DCM may complete pregnancy without major neonatal complications, the risk of fetal and perinatal adverse events remains higher compared to the general population (Table 4).

In the multicenter study published by Restrepo-Córdoba et al., among a total of 83 pregnancies in women carrying DCM-related genetic variants, obstetric or neonatal complications were reported in 14% of cases [50]. These events were significantly more frequent in patients with a phenotypic expression of DCM (10 out of 30 pregnancies) compared to asymptomatic carriers (2 out of 18 pregnancies) [50]. Reported complications included intrauterine growth restriction, prematurity, and the need for emergency cesarean section due to maternal hemodynamic instability. In one case, preeclampsia associated with heart failure was also observed, highlighting the complex interplay between obstetric and cardiac factors.

Additional insight comes from a small but well-characterized cohort that followed seven women with DCM during pregnancy and up to 18 months postpartum: all newborns were delivered at term with normal Apgar scores and umbilical cord pH within the normal range [52]. However, two infants had low birth weight, consistent with literature data reporting an increased incidence of small-for-gestational-age (SGA) newborns in pregnancies complicated by maternal heart disease [52].

In the clinical case reported by Shotan et al., involving a twin pregnancy in a woman with severe DCM and an LVEF < 30%, the newborns had significantly below-average birth weights (1926 g and 1774 g), but normal Apgar scores and umbilical cord pH [6]. This supports the notion that in high-risk settings, fetal growth may be impaired even in the absence of overt obstetric complications, and that fetal growth monitoring should be an integral part of multidisciplinary follow-up.

Other studies have documented an increase in iatrogenic prematurity, driven by the need to deliver early due to maternal instability or onset of HF. Peters et al. describe a case of severe third-trimester heart failure requiring early delivery and mechanical circulatory support [51]. In such settings, neonatal complications may include not only prematurity, but also respiratory distress, hypoglycemia, and the need for neonatal intensive care unit admission.

In conclusion, neonates born to mothers with DCM are at increased risk of adverse outcomes. Regular fetal ultrasound monitoring and a coordinated obstetric plan are essential to optimize perinatal outcomes.

### 5.2. Multidisciplinary Approach, Genetic Implications, and Long-Term Perspectives in the Management of Pregnancy in Dilated Cardiomyopathy

**Figure 4:**
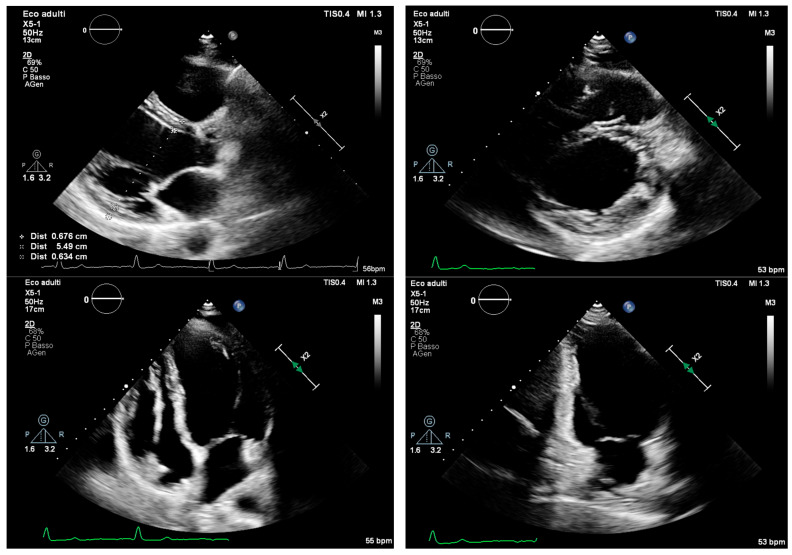
Echocardiographic images of a patient at 38 weeks of gestation affected by dilated cardiomyopathy with left ventricular systolic function at the lower limit of normal. She had a history of syncope and previously documented second-degree atrioventricular block (type 2:1), which alternated with trifascicular block. The patient also experienced episodes of non-sustained ventricular tachycardia and paroxysmal atrial fibrillation. Genetic testing revealed a variant of uncertain significance in the myopalladin gene. Pregnancy did not result in a deterioration of left ventricular systolic function or an increase in ventricular volumes.

The management of pregnancy in patients with DCM requires a multidisciplinary approach involving cardiologists, obstetricians, anesthesiologists, and geneticists, with well-structured preconception planning and postpartum follow-up, as underlined by current guidelines [8] (Figure 4). From an obstetric standpoint, vaginal delivery is generally recommended when clinical conditions allow; however, the rate of cesarean sections increases in cases of acute heart failure or hemodynamic instability. In the data reported by Restrepo-Córdoba et al., 32% of deliveries were performed via cesarean section, with half of these due to cardiac indications [50].

Careful management of hemodynamic load has been recommended during labor and the immediate postpartum period, given the increased risk of decompensation reported in the literature [34,35,36]. The use of epidural anesthesia is preferred to reduce pain and sympathetic activation, but it requires close hemodynamic monitoring and, often, central venous access [6].

The role of genetics in DCM is becoming increasingly central, particularly in the context of reproductive health. Pathogenic variants in genes such as *TTN*, *LMNA*, *FLNC*, and *RBM20* are commonly identified in women of childbearing age and may confer a high risk of developing the phenotype during or after pregnancy. In multicenter data, variants in *TTNtv*, *MYH7*, and *RBM20* were associated with severe events, including sudden cardiac death and advanced heart failure [51].

Among the most relevant genetic findings in pregnancy-related DCM are truncating variants in *TTN* (TTNtv), which are frequently observed both in patients diagnosed during pregnancy and in those with postpartum onset [51]. Some of these variants have been associated with a severe phenotype requiring mechanical circulatory support or resulting in maternal death. Although *LMNA* mutations account for a minority of familial DCM cases, they are particularly noteworthy due to their early onset, high incidence of ventricular arrhythmias, and rapid decline in ventricular function [54,55]. In a Finnish cohort of women carrying *LMNA* mutations, no significant deterioration was observed during pregnancy; however, the accompanying editorial literature warns of the arrhythmic risk, even in seemingly stable carriers [54,55].

Variants in *FLNC*, often linked to more severe phenotypes and a high arrhythmic burden, also require careful risk stratification. Several case series have reported substantial clinical deterioration during pregnancy in carriers of *FLNC* mutations, with the onset of heart failure even in previously asymptomatic women [54]. In this context, the role of genetic counseling is crucial and should be offered to all women with a family history of DCM from the earliest stages of pregnancy planning. Early identification of high-risk mutations enables proactive management, including intensive monitoring during pregnancy, consideration of implantable devices for carriers of *LMNA* or *FLNC* mutations, and discussions regarding assisted reproduction options with preimplantation genetic diagnosis.

Genetic counseling has implications that are not only clinical but also reproductive. Given the autosomal dominant inheritance of most familial DCMs, up to 50% of offspring may inherit the pathogenic variant. It is therefore essential to provide counseling on the risk of transmission, the availability of family screening, and options for preimplantation diagnosis. In this setting, a multidisciplinary cardio-genetic team now represents the standard of care for women with hereditary DCM who wish to pursue pregnancy.

Postpartum follow-up represents a critical yet often underestimated phase, as severe cardiac events, including heart failure and arrhythmias, can occur up to six months after delivery. In a subset of the study by Restrepo-Córdoba, late adverse events were observed, including deterioration of ventricular function, even among women who had tolerated pregnancy well [50].

Serial measurement of NT-proBNP levels may be useful in the early identification of clinical deterioration [52,53]. Furthermore, long-term reproductive implications should be addressed from the time of preconception counseling: the potential for structural and functional worsening, the risk of genetic transmission, and the need for effective contraception, preferably intrauterine devices in high-risk cases, should be discussed in a shared decision-making process with the patient.

## 6. Arrhythmogenic Cardiomyopathy in Pregnancy: Maternal Risks, Obstetric Outcomes, and Disease Progression

**Figure 5:**
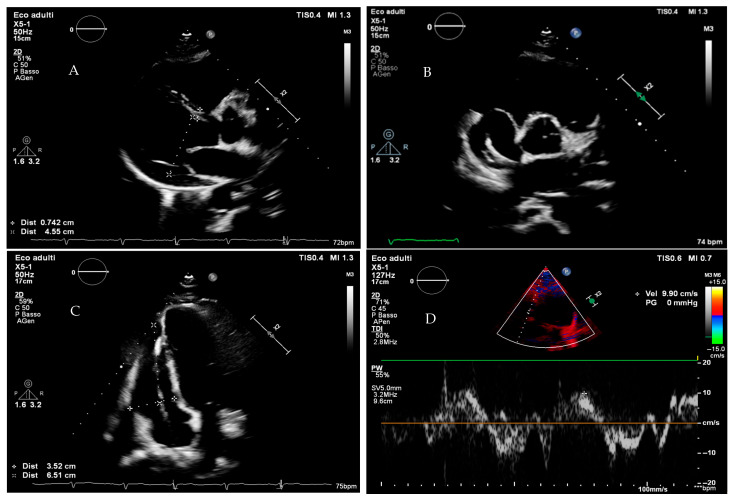
Transthoracic echocardiographic images from a woman who presented with sustained ventricular tachycardia during pregnancy. Subsequent evaluation revealed a pathogenic variant in a desmosomal gene, leading to the diagnosis of arrhythmogenic cardiomyopathy. Left ventricular systolic function is preserved. (**Panel A**): Parasternal long-axis view showing normal left ventricular wall thickness and chamber dimensions. (**Panel B**): Parasternal short-axis view showing right ventricular dilation. (**Panel C**): The right ventricle appears mildly dilated (The line between the asterisks), with reduced tissue Doppler velocities (**Panel D**).

ARVC, most commonly linked to mutations in desmosomal genes, is a myocardial disorder predisposing to ventricular arrhythmias (VAs) and sudden cardiac death (SCD) [56,57,58]. It poses a distinct challenge during pregnancy. While the hormonal and hemodynamic changes of gestation might theoretically exacerbate arrhythmic risk or accelerate myocardial remodeling, real-world data paint a more nuanced picture (Figure 5).

One of the most comprehensive contributions comes from the Johns Hopkins–Dutch ARVD/C registry, which followed 26 women across 39 singleton pregnancies [59]. The findings were largely reassuring: only 13% experienced sustained VAs, and 5% developed decompensated heart failure. All events occurred in women with known structural disease or arrhythmic history. Importantly, no maternal mortality was reported, and all children were live-born without major obstetric complications. Cesarean section was performed in 28% of cases, primarily for obstetric reasons. Notably, beta-blocker therapy was associated with a significant reduction in birth weight (3.1 ± 0.48 kg vs. 3.7 ± 0.57 kg; *p* = 0.002) [59].

The Nordic ARVC Registry, encompassing 199 women (121 with definite ARVC and 78 mutation-positive relatives), confirmed these reassuring findings [60]. Among 261 pregnancies, only two arrhythmic events occurred during gestation, and the postpartum period (especially year one) appeared more vulnerable, with a hazard ratio of 13.7 for VA events in women who conceived after diagnosis [60]. The Nordic registry found no difference in VA-free survival or disease progression between women with 1, 2, or ≥3 childbirths. Still, the absolute event rate remained low, and no maternal deaths or peripartum SCDs were observed.

Further validation comes from Castrini et al., who assessed 77 women over a median of 19 years post-pregnancy [61]. Despite some reports of palpitations and mild symptoms during pregnancy, there was no association between the number of pregnancies and the severity of ARVC, arrhythmic events, or decline in ventricular function [61]. The authors concluded that pregnancy did not appear to accelerate disease progression, even in women with ≥3 deliveries. Smaller series and case reports mirror these observations. In a Canadian cohort of nine women, all tolerated pregnancy well; five had ICDs, and none experienced decompensated heart failure [62]. All neonates were live-born, with a 33% rate of SGA births. Breastfeeding was feasible in all cases, and beta-blockers were maintained without incident [62]. A study of 224 pregnancies in 157 women reported a 5.4% rate of cardiac events, mostly in those with lower baseline LVEF [63]. Women with preserved function and no arrhythmic history had uneventful gestations [63].

These data collectively suggest that pregnancy is generally well tolerated in women with ARVC, particularly when ventricular function is preserved, and arrhythmia burden is stable (Table 5). However, arrhythmic events, though infrequent, tend to occur in women with prior history of sustained VT or ICD therapies, highlighting the importance of preconception risk stratification and continuous beta-blockade when indicated. Moreover, the postpartum period appears to carry the highest risk, necessitating close surveillance beyond delivery.

### Postpartum Management in Women with ARVC

The postpartum period represents a particularly vulnerable window for women with ARVC, even more so than pregnancy itself. Evidence from registries suggests a marked increase in arrhythmic burden during the first 6–12 months after delivery.

In the Nordic ARVC Registry, women who conceived after their ARVC diagnosis experienced a 13.7-fold higher risk of ventricular arrhythmias during the first year postpartum compared to the non-pregnant period [60]. Most events occurred within the first 6 months and were more common in women with prior sustained VT or ICD therapies, suggesting that hormonal shifts, autonomic instability, and volume fluctuations in the postpartum period may act as arrhythmogenic triggers [60]. Castrini et al. observed similar trends: while pregnancy itself did not accelerate disease progression, a minority of patients experienced new or worsened arrhythmias postpartum [61]. These events did not correlate with the number of pregnancies, suggesting that individual arrhythmic substrate and genetic background play a larger role than parity in determining long-term prognosis [61]. Importantly, the number of pregnancies does not appear to adversely impact long-term disease severity in ARVC. In both the Nordic Registry and the Johns Hopkins cohort, no correlation was found between parity and progression of ventricular dysfunction, arrhythmia burden, or need for advanced therapies [59,60]. This allows for more flexible reproductive planning in clinically stable women.

Postpartum management should include close rhythm surveillance, especially in the first 6 months. For women with prior sustained VT or syncope, continuous or event-triggered ECG monitoring, titration of beta-blockers, and reinforcement of ICD programming are essential [64]. In cases of new-onset arrhythmia, early electrophysiology consultation is recommended, with consideration of catheter ablation if burden is high or symptomatic.

## 7. Restrictive, Infiltrative, and Inflammatory Cardiomyopathies in Pregnancy: Rare Entities with High-Stakes Implications

RCM, infiltrative diseases such as cardiac amyloidosis, and inflammatory forms like sarcoidosis and autoimmune myocarditis represent rare but clinically challenging scenarios when encountered during pregnancy. Due to their low prevalence and diagnostic complexity, these phenotypes are often underrepresented in cardio-obstetric literature [1]. Nevertheless, selected case reports and registry-based studies provide valuable insight into maternal-fetal risk and management strategies (Table 6).

RCM is arguably the least studied among the primary cardiomyopathies in pregnancy. Its hallmark features (marked diastolic dysfunction, preserved systolic function, and biatrial enlargement) are poorly tolerated in the volume-expanded state of gestation [1]. While no prospective data exist, retrospective reports indicate that women with RCM and elevated filling pressures may experience pulmonary edema and atrial arrhythmias as early as the second trimester [15,65,66,67].

Infiltrative cardiomyopathies, particularly cardiac amyloidosis, are exceedingly rare in women of reproductive age. However, case reports suggest that hereditary transthyretin (TTR) mutations may be encountered earlier in life than wild-type forms [10,33].

Cardiac sarcoidosis and inflammatory cardiomyopathies pose diagnostic and prognostic dilemmas in pregnancy due to their arrhythmogenic potential and need for immunosuppression. Patients typically present with conduction abnormalities, non-sustained ventricular tachycardia, or patchy late gadolinium enhancement on cardiac MRI. Diagnosis is often delayed until the postpartum period, when advanced imaging and histological assessment become feasible.

Pregnancy can unmask latent inflammatory disease or exacerbate active myocarditis. Although high-dose corticosteroids may be necessary in severe presentations, their use during pregnancy must be balanced against fetal risk. In reported cases, patients with known sarcoidosis and cardiac involvement required tapered steroid regimens and rhythm monitoring, with some requiring ICD implantation postpartum due to persistent ventricular arrhythmias.

The postpartum period appears particularly high risk across all these phenotypes. Cases of sudden decompensation, new-onset ventricular dysfunction, or arrhythmic storm have been described, likely due to abrupt hormonal shifts and immune reconstitution. Hence, early postpartum follow-up, echocardiography, and rhythm surveillance are essential, even in women who were stable during gestation.

## 8. Lactation in the Context of Cardiomyopathy: Between Caution and Clinical Evidence

Breastfeeding is widely recognized for its substantial benefits to both maternal and neonatal health, encompassing improved immunological protection, metabolic regulation, psychological bonding, and long-term reductions in maternal cardiovascular and oncological risks. Nevertheless, in the setting of cardiomyopathies, and particularly in peripartum cardiomyopathy (PPCM), the decision to initiate or continue breastfeeding is often fraught with clinical uncertainty. Historically, concerns have revolved around two main issues: the potential pathophysiological role of prolactin in PPCM and the lack of robust data on the safety of heart failure pharmacotherapy during lactation [4].

The hypothesis linking prolactin to PPCM pathogenesis was first supported by animal studies suggesting that a cleaved 16-kDa fragment of prolactin may exert vasculotoxic effects, contributing to microvascular dysfunction and cardiomyocyte injury. Based on this theory, prolactin inhibition, most achieved with dopamine agonists such as bromocriptine, has been proposed as a disease-modifying strategy [2,4]. However, clinical evidence remains limited and somewhat inconsistent. While some European studies have shown favorable effects on recovery with bromocriptine, these results stem from small cohorts and lack long-term follow-up. Moreover, bromocriptine suppresses lactation, introducing an immediate trade-off between potential maternal benefit and the recognized nutritional and immunological advantages of breastfeeding.

Recent prospective data from the U.S.-based IPAC study (Investigations of Pregnancy-Associated Cardiomyopathy) have helped to reshape this narrative [68]. In this multicenter study of 100 women with newly diagnosed PPCM, no statistically significant difference in LVEF recovery was observed between women who breastfed and those who did not, despite higher serum prolactin levels and increased CD8+ cytotoxic T cell activation in the breastfeeding group [68]. This challenges the presumed deleterious role of prolactin in PPCM and raises important questions about the generalizability of animal data to human populations. Notably, the observed immune activation did not translate into worse clinical outcomes, suggesting that prolactin-related immune shifts may not be intrinsically harmful.

Similarly, a retrospective study conducted at the University of Michigan found no association between breastfeeding and impaired myocardial recovery in a well-characterized cohort of PPCM patients. Even among women with severely reduced LVEF at presentation (≤30%), breastfeeding was not linked to worse outcomes [69]. Yet, more than 60% of surveyed patients reported being explicitly discouraged from breastfeeding due to their diagnosis or medication regimens, and none recalled receiving active encouragement to breastfeed [69]. This underscores a dissonance between emerging evidence and real-world counseling practices, potentially depriving many women and their infants of the benefits of breastfeeding without sufficient justification.

From a pharmacological perspective, concerns regarding the transfer of heart failure medications into breast milk are valid but, in many cases, overstated. Most prescribed agents, including beta-blockers, ACE inhibitors (notably enalapril), loop and thiazide diuretics, hydralazine, and nitrates, demonstrate limited excretion into breast milk and are generally considered compatible with lactation when appropriately monitored [70,71]. For –8/instance, enalapril is not only associated with minimal milk-to-plasma ratios, but is also used therapeutically in neonates, providing additional reassurance [70]. Loop diuretics, while having the potential to reduce milk supply in high doses, have poor oral bioavailability in neonates and are widely used with caution [70]. That said, specific agents such as milrinone and levosimendan warrant greater caution due to their pharmacokinetic profiles and potential accumulation in breastfed infants [70]. For these agents, temporary interruption of breastfeeding and milk expression with discarding may be advisable. Moreover, during acute decompensation requiring inotropes or intravenous vasodilators, the feasibility of breastfeeding may also be limited by maternal clinical status alone.

Current guidelines remain cautious. The European Society of Cardiology (ESC) working group on PPCM has suggested that breastfeeding may be discouraged in cases of severe cardiac dysfunction, particularly when bromocriptine is employed, but may be considered in women with mild dysfunction and stable clinical profiles [3]. However, these recommendations are based largely on pathophysiological assumptions and expert opinion rather than high-level evidence.

Given the growing body of observational and prospective data suggesting no harm, and potentially some benefit, from breastfeeding in stable women with PPCM and cardiomyopathies, several authors have proposed a more nuanced and individualized approach. Clinical decisions should involve shared decision-making and consider maternal hemodynamic status, pharmacotherapy, infant prematurity, and maternal preferences. Counseling should shift from a default stance of discouragement toward informed discussion, risk stratification, and support for breastfeeding where appropriate.

Finally, the lack of prospective randomized trials in this domain remains a major limitation. Future cohort studies should prioritize the systematic collection of breastfeeding data, including duration, exclusivity, and infant outcomes. Until then, clinicians must balance the known maternal and infant benefits of breastfeeding with the individualized assessment of cardiovascular risk and treatment exposure.

## 9. Contraceptive Counseling in Women with Cardiomyopathies: A Tailored Approach

Effective contraceptive counseling is an essential component of care in women with cardiomyopathies, given the high maternal and fetal risks associated with unplanned pregnancies, particularly in those with moderate-to-severe ventricular dysfunction or advanced symptomatic status. The choice of contraceptive method must be individualized, accounting for both the cardiovascular profile and comorbid risk factors.

Among women with DCM or severe left ventricular systolic dysfunction (LVEF < 30%, NYHA class III–IV), pregnancy is considered extremely high risk (mWHO IV), and all efforts should be made to avoid unplanned conception. In such cases, intrauterine devices (IUDs), especially the levonorgestrel-releasing type, are the preferred option due to their high efficacy and safety profile [7]. Copper IUDs may be less suitable in patients on anticoagulation or with menorrhagia [7]. Progestin-only pills or implants represent valid alternatives, while combined estrogen-progestin formulations are generally contraindicated in patients with heart failure or atrial fibrillation due to increased thromboembolic risk [9].

In HCM, the overall tolerance to pregnancy is better, although women with LVOTO, prior arrhythmias, or NYHA class ≥ II remain at increased risk. In these patients, estrogen-containing contraceptives should be used with caution, especially in the presence of atrial arrhythmias or prior embolic events [14]. Progestin-only contraception and IUDs are generally safe and preferred [14]. The use of estrogen-containing pills may be acceptable only in those with asymptomatic HCM and no history of arrhythmia [7].

In ARVC, the main concern is the risk of ventricular arrhythmias during pregnancy and postpartum. Although pregnancy is often tolerated, the arrhythmic burden in probands or those with biventricular involvement necessitates effective contraception. Hormonal IUDs and progestin-only pills are preferred, while combined hormonal contraceptives are generally avoided due to arrhythmic and thrombotic concerns [7,11].

In agreement with the gynecologist, a thrombophilia screening should be considered prior to initiating hormonal contraception, particularly in patients with a personal or family history of thromboembolic events.

Across all cardiomyopathy subtypes, shared decision-making, multidisciplinary follow-up, and timely initiation of contraception are essential to minimize risk and guide safe reproductive planning.

## 10. The Cardio-Obstetric Practice: What Lies Ahead?

In 2021, the Italian National Institute of Health issued guidelines outlining best practices for the clinical management of pregnancy and the postpartum period in women affected by congenital cardiomyopathies [72]. These recommendations emphasize the necessity of a multidisciplinary approach for women with moderate to high-risk cardiovascular conditions. The recommended care team includes cardiologists, maternal-fetal medicine (MFM) specialists, neonatologists, anesthesiologists, and midwives experienced in managing high-risk pregnancies.

The risk-adapted management model described in the guidelines clearly delineates the roles of different healthcare levels. Women classified as low-risk may be safely managed in primary-level obstetric centers, which offer basic medical and surgical services and are adequately equipped to handle uncomplicated pregnancies. Conversely, secondary-level centers—referral hospitals with access to advanced medical specialties such as interventional cardiology, cardiothoracic surgery, neurosurgery, and neonatal intensive care—are designated for the management of complex and high-risk cases [73].

Women identified as being at moderate risk are ideally managed in secondary-level obstetric centers throughout pregnancy. In situations where geographic constraints prevent continuous care at such centers, these women may be followed at a primary-level facility with the stipulation that they undergo multidisciplinary evaluation—including consultation with a cardiologist and an MFM specialist—once per trimester at a secondary-level center. High-risk patients, on the other hand, require comprehensive prenatal and peripartum management exclusively in secondary-level obstetric facilities and dedicated adult cardiology referral centers. This reinforces the critical importance of a highly specialized, multidisciplinary cardio-obstetric team.

In the United States, several institutions are pioneering structured cardio-obstetric programs. For example, Brigham and Women’s Hospital in Boston offers a comprehensive care model that integrates cardiovascular and MFM expertise for high-risk pregnancies. Similarly, the University of Rochester Medical Center in New York has established a dedicated cardio-obstetric care line that includes postpartum surveillance and preconception counseling for women with structural or acquired cardiac disease [74].

Nevertheless, the implementation of integrated cardio-obstetric care in the United States remains fragmented [74]. Only 10% of surveyed cardiologists reported participation in structured programs, and substantial knowledge gaps persist regarding pharmacologic safety, management of valvular disease, and acute coronary syndromes during pregnancy and lactation.

In Italy, care programs for women with congenital heart disease exist in both public and private healthcare institutions. However, these institutions often serve as centers of excellence and typically provide care to patients referred by other physicians.

There is limited information regarding the existence of structured, institution-wide care pathways specifically dedicated to women with cardiomyopathies. Furthermore, the preconception phase—a critical period for the management of women with known or suspected cardiac conditions—is often neglected. Recent literature underscores the need for a collaborative care model that includes primary care providers, who are typically the first point of contact for many women [75,76].

Postnatal care represents another major gap in the continuum of cardio-obstetric care. In Italy, this responsibility primarily falls to the *consultorio*—a community-based health service designed to provide comprehensive care for women during pregnancy and the puerperium. However, service availability is unevenly distributed across the country, with significantly greater offer in northern regions compared to the south. National data indicate that only 35% of pregnant women access *consultori* services at least once, suggesting that continuous postpartum follow-up is even less common [77]. For women with cardiomyopathies, effective postnatal monitoring is essential. The American College of Obstetricians and Gynecologists (ACOG) recommends a postpartum follow-up within 7–14 days for women with cardiovascular disease. Yet, little is known about whether such standards are being implemented in Italian primary or secondary care settings.

There remains a significant gap in the mapping and development of dedicated, structured programs for pregnant women with cardiomyopathies within the Italian healthcare system. Identifying and cataloging existing programs could serve as an essential first step in scaling up services nationwide.

Nonetheless, establishing such programs alone is insufficient. A truly effective care model must extend across the entire perinatal spectrum—from preconception to postpartum—and involve both primary and secondary care providers. An integrated, longitudinal care model encompassing general practitioners, pediatricians, specialized cardio-obstetric teams, and *consultori* would represent a more equitable and comprehensive solution.

Finally, issues of territorial inequity must be addressed. High-quality care should not be confined to specific regions or elite institutions but must be accessible across the entire country. Ensuring equitable access to care—regardless of socioeconomic status or geographic location—is not merely a public health priority but a matter of reproductive and social justice.

## 11. Limitations

This review presents several limitations inherent to the scoping review methodology. First, no formal quality assessment or risk of bias appraisal of included studies was performed, as the primary aim was to map the available evidence rather than to provide a quantitative synthesis. Second, the heterogeneity of study designs, populations, and outcome definitions across the included literature limits the comparability of findings and precludes meta-analytic conclusions. Third, the majority of available data derive from retrospective cohorts, case series, or expert consensus, with a relative paucity of prospective studies specifically focused on women of reproductive age with cardiomyopathies. Fourth, given the narrative nature of this review, some sections integrate evidence-based findings with expert interpretation. We have aimed to clarify where recommendations reflect published guidelines or primary data versus clinical opinion. Finally, despite an extensive literature search across three major databases, some relevant studies may have been missed due to publication bias, language restrictions, or incomplete indexing. Nevertheless, this review offers a comprehensive and updated overview of current knowledge and clinical challenges, identifying key gaps and priorities for future research.

## 12. Conclusions

Pregnancy in women with cardiomyopathies poses a complex clinical challenge, requiring careful balance between maternal safety and fetal well-being. Although many women with stable disease and preserved function can complete pregnancy without major complications, others face significant risks, especially during the third trimester and early postpartum. The type and severity of cardiomyopathy, genetic background, previous cardiac events, and hemodynamic reserve are all key determinants of outcome.

Early multidisciplinary assessment and shared decision-making remain the cornerstone of care. As diagnostic technologies and therapeutic options evolve, the identification of genotype-phenotype correlations and the development of personalized management pathways will be essential.

Finally, reproductive counseling, including contraceptive planning and breastfeeding support, must be integrated into routine care for women with cardiomyopathy, ideally beginning in adolescence or early adulthood. The growing field of cardio-obstetrics offers a unique opportunity to translate evidence into practice and close long-standing gaps in gender-specific cardiovascular care.

## Figures and Tables

**Table 1:** Cardiomyopathy and pregnancy risk overview.

Cardiomyopathy	Maternal Risk	Fetal Risk	Critical Window	Typical Maternal Age Range	Allowed Medications
DCM	High	Moderate-High	3rd trimester, postpartum	20–40 years	BB, diuretics, ACEi postpartum
		(risk of familial DCM in fetus if genetic form)			
HCM	Moderate	Low (possible inherited HCM phenotype in fetus)	Labor, postpartum	20–40 years	BB, +/− Verapamil
ARVC	Low-Moderate	Low (risk of ARVC phenotype in familial forms)	Postpartum	Typically < 40 years	BB, arrhythmia control
RCM	High	High (familial/genetic forms possible)	Throughout pregnancy	Variable	Individualized therapy

ARVC = Arrhythmogenic Right Ventricular Cardiomyopathy; DCM = Dilated Cardiomyopathy; HCM = Hypertrophic Cardiomyopathy; RCM = Restrictive Cardiomyopathy; BB = Beta-Blocker; ACEi = Angiotensin-Converting Enzyme inhibitor.

**Table 2:** Comparison of risk stratification models for pregnancy in women with cardiomyopathy.

Score	Population of Origin	Main Variables	Strengths	Limitations in CMP
mWHO	Expert consensus (ESC)	Underlying diagnosis (e.g., DCM, HCM, ARVC)	Simple to apply; disease-based; endorsed by ESC	Is not sufficiently specific; does not capture individual variability
CARPREG I	Mixed CV disease (2001)	NYHA class > II, prior cardiac events, arrhythmias, LV obstruction, cyanosis	Validated; practical	Limited representation of cardiomyopathies; static model
CARPREG II	Multicenter registry, expanded cohort (2018)	Adds LVEF < 40%, mechanical valve, anticoagulation, severe valvular lesions	Better discrimination in CMP; echocardiographic integration	Still underperforms in rare or genotype-driven cardiomyopathies
ZAHARA	Women with congenital heart disease (Dutch registry)	Includes arrhythmias, prior events, medication, LVEF, left heart obstruction	Comprehensive; includes fetal risk	Low applicability in CMP; poorer discrimination in non-congenital heart diseases

ARVC = arrhythmogenic right ventricular cardiomyopathy; CARPREG = CARdiac disease in PREGnancy; CMP = cardiomyopathy; CV = cardiovascular; DCM = dilated cardiomyopathy; ESC = European Society of Cardiology; HCM = hypertrophic cardiomyopathy; LV = left ventricle; LVEF = left ventricular ejection fraction; mWHO = modified WHO; NYHA = New York Heart Association functional class; ZAHARA = Zwangerschap bij Aangeboren HARtAfwijkingen.

**Table 3:** Pregnancy outcomes in women with hypertrophic cardiomyopathy: Key Studies.

Study	N°. of Patients	HCM Type	MACE (%)	Identified Risk Factors	Notes
Fumagalli et al. [34]	242 women (432 pregnancies)	Mixed	<8%	NYHA ≥ II, known diagnosis before pregnancy	Pregnancy associated with reduced CV risk
ROPAC Registry (Goland et al.) [35]	60	42% OHCM	23%	HF, arrhythmias; clustered in late pregnancy and postpartum	No maternal deaths
Choi et al. (Korea) [36]	122	Not specified	8.8%	Pre-existing AF, VT (OR > 7)	Nationwide population-based study
Huang et al. (China) [37]	100	OHCM vs. NOHCM	9%	OHCM, NYHA ≥ II, syncope	2% maternal mortality
Alves-Pinto (Case Report) [38]	1	Severe OHCM (LVOTO 90 mmHg)	—	—	Preterm delivery at 34 weeks due to fetal growth restriction

AF = Atrial Fibrillation; LVOTO = Left Ventricular Outflow Tract Obstruction; MACE = Major Adverse Cardiovascular Events; NYHA = New York Heart Association functional class; NOHCM = Non-Obstructive HCM; OHCM = Obstructive HCM; VT = Ventricular Tachycardia.

**Table 4:** Fetal and neonatal outcomes in pregnancies with maternal DCM.

Scheme	Total Pregnancies	Fetal/Neonatal Events (%)	Main Events
Restrepo-Córdoba et al. [50]	83	14%	IUGR, prematurity, emergency C-section, 1 preeclampsia + HF
Blatt et al. [52]	7	28.6%	2 low birth weight infants
Shotan et al. (case) [6]	1 twin pregnancy	—	Both SGA, normal Apgar/pH
Peters et al. [51]	Not specified	Not specified	Preterm delivery, NICU admission

Apgar = Apgar Score; DCM = Dilated Cardiomyopathy; IUGR = Intrauterine Growth Restriction; NICU = Neonatal Intensive Care Unit; pH = Umbilical Arterial pH; SGA = Small for Gestational Age. Note: Listed fetal/neonatal events arise from maternal cardiomyopathy complications, not from inherited fetal cardiomyopathy phenotypes.

**Table 5:** Summary of studies on arrhythmogenic cardiomyopathy and pregnancy.

Study	No. of Women/Pregnancies	Adverse Events	Neonatal Outcomes	Comments
Johns Hopkins–Dutch Registry [59]	26/39	13% VAs, 5% HF; only in patients with known disease history	All live births, no major complications; reduced birth weight with beta-blockers	All events in patients with prior disease; no maternal deaths
Nordic ARVC Registry [13]	199/261	2 arrhythmic events during pregnancy; HR 13.7 for VAs postpartum	No maternal or neonatal deaths	Postpartum period highest risk; parity not linked to worse outcomes
Castrini et al. [61]	77/144	No pregnancy-related worsening of disease severity	Breastfeeding feasible in all; no critical neonatal events	Long-term follow-up; stable disease course
Luo et al. [62]	9/9	0 HF; 33% SGA births	All live births, 33% small-for-gestational-age	ICDs well tolerated; beta-blockers maintained during pregnancy
Wu et al. [63]	157/224	5.4% cardiac events, mostly in patients with reduced LVEF	No major neonatal complications reported	Favorable outcomes with preserved ventricular function

ARVC = Arrhythmogenic right ventricular cardiomyopathy; HF = Heart failure; HR = Hazard ratio; ICD = Implantable cardioverter defibrillator; LVEF = Left ventricular ejection fraction; SGA = Small-for-gestational-age; VA = Ventricular arrhythmia; VT = Ventricular tachycardia.

**Table 6:** Summary of rare cardiomyopathies in pregnancy.

Phenotype	Key Characteristics	Reported Complications	Postpartum Considerations
Restrictive Cardiomyopathy (RCM)	Diastolic dysfunction, biatrial enlargement, preserved EF	Pulmonary edema, atrial arrhythmias (2nd trimester)	High risk for decompensation, need for early echo and rhythm follow-up
Cardiac Amyloidosis (ATTR)	Transthyretin mutations, early onset possible in hereditary forms	No large series; possible early onset symptoms in hereditary cases	Unclear data; vigilance recommended due to amyloid progression
Cardiac Sarcoidosis	Patchy LGE on MRI, conduction disease, arrhythmias	Sustained VT, need for steroids, ICD postpartum	ICD implantation often required; rhythm instability
82399/	Inflammatory, often delayed diagnosis, arrhythmic potential	Arrhythmic storms, immunosuppression needs, delayed diagnosis	High-risk period, immune rebound, monitor for new/worsened symptoms

ATTR = Transthyretin amyloidosis; EF = Ejection fraction; ICD = Implantable cardioverter defibrillator; LGE = Late gadolinium enhancement; RCM = Restrictive cardiomyopathy; VT = Ventricular tachycardia.

## Data Availability

Data sharing not applicable.

## Abbreviations

The following abbreviations are used in this manuscript:

ACOG	American College of Obstetricians and Gynecologists
ARCV	arrhythmogenic right ventricular cardiomyopathy
ASA	Alcohol septal ablation
CARPREG	CARdiac disease in PREGnancy;
CMP	cardiomyopathy
CV	cardiovascular
DCM	dilated cardiomyopathy
ESC	European Society of Cardiology
*FLNC*	Filamin C
HCM	hypertrophic cardiomyopathy
ICD	implantable cardioverter defibrillator
IUDs	Intrauterine devices
*LMNA* gene	Lamin A/C gene
LV	left ventricle
LVEF	left ventricular ejection fraction
LVOTO	left ventricular outflow tract obstruction
MACE	Major adverse cardiovascular events
MFM	Maternal-fetal medicine
mWHO	modified World Health Organization
NOHCM	Non-obstructive hypertrophic cardiomyopathy
NYHA	New York Heart Association functional class
NT-proBNP	N-terminal pro–B-type natriuretic peptide
OHCM	Obstructive hypertrophic cardiomyopathy
PPCM	postpartum cardiomyopathy
*RBM20*	RNA-binding motif protein 20 gene
RCM	restrictive cardiomyopathy
ROPAC registry	Registry of Pregnancy and Cardiac Disease
SCD	sudden cardiac death
SGA	small-for-gestational-age
SRT	Septal reduction therapy
TMEM43	transmembrane protein 43
TTN	Titin gene
TTNtv	truncating variants in *TTN* gene
TTR	transthyretin
VAs	Ventricular arrhythmias
WOS	Web of science
ZAHARA	Zwangerschap bij Aangeboren HARtAfwijkingen
